# Strengthening Addiction Care Continuum Through Community Consortium in Vietnam: Protocol for a Cluster-Randomized Controlled Trial

**DOI:** 10.2196/44219

**Published:** 2023-03-22

**Authors:** Li Li, Tuan Anh Nguyen, Li-Jung Liang, Chunqing Lin, Thang Hong Pham, Ha Thi Thanh Nguyen, Steven Kha

**Affiliations:** 1 University of California, Los Angeles Los Angeles, CA United States; 2 National Institute of Hygiene and Epidemiology Hanoi Vietnam

**Keywords:** addiction service, community consortium, drug addiction, health care worker, health, family, Vietnam

## Abstract

**Background:**

A chronic condition, drug addiction, requires long-term multipronged health care and treatment services. Community-based approaches can offer the advantages of managing integrated care along the care continuum and improving clinical outcomes. However, scant rigorous research focuses on sustainable, community-based care and service delivery.

**Objective:**

This protocol describes a study aiming to develop and test an intervention that features the alliance of community health workers and family members to provide integrated support and individualized services and treatment for people who use drugs (PWUD) in community settings.

**Methods:**

Based on the National Institute on Drug Abuse’s Seek-Test-Treat-Retain (STTR) framework, an intervention that provides training to community health workers will be developed and piloted before an intervention trial. Trained community health workers will conduct home visits and provide support for PWUD and their families. The intervention trial will be conducted in 3 regions in Vietnam, with 60 communities (named communes). These communes will be randomized to either an intervention or control condition. Intervention outcomes will be evaluated at baseline and at 3, 6, 9, and 12 months. The primary outcome measure is PWUD’s STTR fulfillment, consisting of multiple individual fulfillment indicators across 5 domains: Seek, Test, Treat, Retain, and Health. The secondary outcomes of interest are the community health workers’ service provision and family members’ support. The primary analysis will follow an intention-to-treat approach. Generalized mixed-effects regression models will be used to compare changes in the outcome measures from baseline between intervention and control conditions.

**Results:**

During the first year of the project, we conducted formative studies, including in-depth interviews and focus groups, to identify service barriers and intervention strategies. The intervention and assessment pilots are scheduled in 2023 before commencing the trial. Reports based on the baseline data will be distributed in early 2024. The intervention outcome results will be available within 6 months of the final data collection date, that is, the main study findings are expected to be available in early 2026.

**Conclusions:**

This study will inform the establishment of community health workers and family members alliance, a locally available infrastructure, to support addiction services and care for PWUD. The methodology, findings, and lessons learned are expected to shed light on the addiction service continuum’s implementation and demonstrate a community-based addiction service delivery model that can be transferable to other countries.

**Trial Registration:**

ClinicalTrials.gov NCT05315492; https://clinicaltrials.gov/ct2/show/NCT05315492

**International Registered Report Identifier (IRRID):**

DERR1-10.2196/44219

## Introduction

Drug addiction, such as opioid use disorder, is one of the most significant public health issues globally [1]. Medication-assisted therapies (MAT), including methadone and buprenorphine or naloxone, are in place to prevent opioid-related overdose and the spread of infectious diseases [2-5]. However, substantial barriers exist to the provision and use of MAT because of the shortage of addiction specialists to prescribe and monitor MAT, especially in remote areas [6,7]. For example, community MAT programs require community members’ support and resources to train community health care workers [6,7].

Mobilization of community-based systems in the service delivery model has been advocated as a desirable strategy to reduce the current gap in MAT [8-12]. In addition, drug addiction, as a chronic condition, requires long-term multipronged health care [13,14]. Community-based approaches can offer the advantages of managing integrated care along the care continuum and improving clinical outcomes [15-18]. However, there is a lack of research focusing on the systematic implementation of MAT and its related service delivery in community care settings [18].

Commune Health Centers (CHCs) are grassroots health care facilities in rural Vietnam, where essential preventive care, initial diagnosis, and referral services can be accessed by residents [19]. Vietnam is one of the first countries to decentralize MAT delivery from centralized treatment sites to community settings [20,21]. However, its CHC-based MAT programs face challenges, such as community health workers (CHWs) lack of capacity in treatment services, misconceptions of harm reduction, and stigmatizing attitudes toward PWUD [22]. Hence, adherence to MAT has been suboptimal, and the patient dropout rates are alarmingly high [23,24].

In Vietnam, the family presents a critical source of support for people who use drugs (PWUD) [25-27]. Families are commonly the source of daily care and financial, emotional, tangible, and informational support for opioid users’ care seeking. The support given by family members can significantly impact the health care use and outcomes of PWUD [28,29]. At the same time, close family members of PWUD often struggle with mental health issues, impaired social adjustment, poor work relations, decreased social participation, and increased intrafamily dysfunction [30,31]. These challenges have jeopardized the family’s capacity to support PWUD in care seeking and adherence.

Current health care infrastructure and culture in Vietnam provide an opportunity to test a community-based service delivery model for addiction care and treatment. We plan to develop and test an intervention that features the alliance of community health workers and family members to provide integrated support and individualized treatment services for PWUD in community settings.

## Methods

### Theoretical Framework

**Figure 1:**
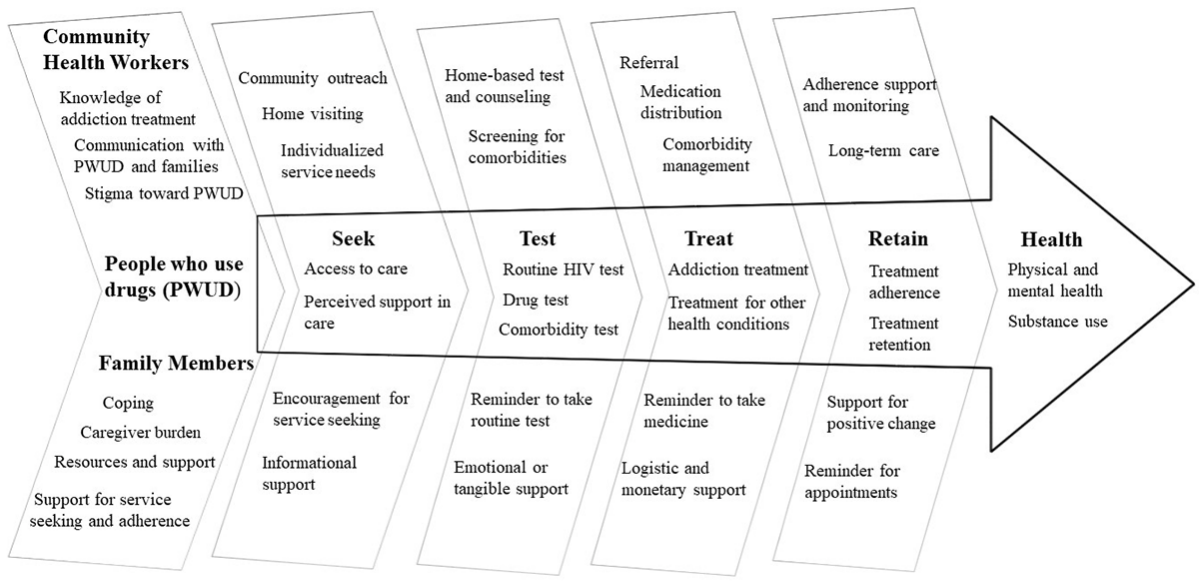
Theoretical Framework.

The National Institute on Drug Abuse’s Seek-Test-Treat-Retain (STTR) initiative provides a framework to guide the delivery of addiction services [32,33]. This approach requires reaching out to PWUD and understanding their health needs (Seek), engaging them in routine physical examinations and laboratory testing (Test), initiating MAT and treatment of comorbidities (Treat), and facilitating treatment adherence and long-term, uninterrupted care (Retain). Based on the theoretical framework, we plan to synergize the support from CHWs and family members as two of the most essential enabling factors to build a respectful, accepting, and supportive addiction care continuum for PWUD (Figure 1). Specifically, CHWs will be trained to provide individualized care with a nonstigmatizing attitude. In addition, family members will be mobilized to give continuous support to PWUD’s service seeking and adherence. The coalition between CHWs and family members will provide a locally available infrastructure to support addiction treatment services, resulting in effective and sustainable outcomes.

### Intervention Development and Pilot

Formative studies, including focus groups and in-depth interviews, will inform intervention development and implementation plan. A total of 3 CHW focus groups and 3 focus groups with community representatives focus on identifying service gaps and effective local strategies to address the challenges. In-depth interviews with PWUD and family members will identify the needs in support, barriers, and facilitators to maintaining service engagement and retention. Following the formative studies, a multidisciplinary work group will be formed and go through 3 steps: (1) reviewing the problem and identifying intervention outcomes, (2) adapting evidence-based strategies to local infrastructure and culture, and (3) developing the intervention implementation and evaluation plan. A pilot version of the intervention program will be produced and followed by an intervention pilot study. The pilot will include a full implementation of intervention activities. In-depth interviews with participants and intervention team members will be conducted to refine the intervention. We will also finalize recruitment procedures and assessment batteries in this phase.

### Cluster-Randomized Controlled Trial

#### Study Design

**Figure 2:**
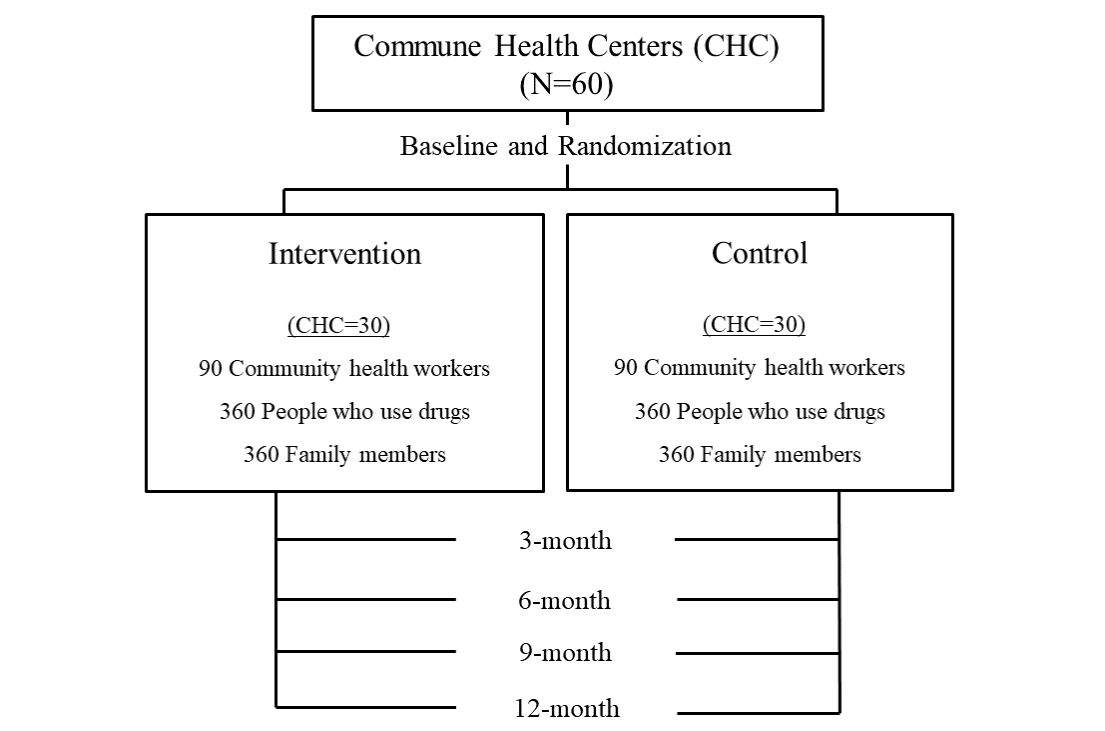
Study design.

The intervention trial aims to test the efficacy of the intervention, primarily with PWUD outcomes, and secondly, at family members and CHW levels. We hypothesize that (1) the intervention will improve PWUD’s service fulfillment along the addiction service continuum, as well as their health outcomes; (2) the intervention will enhance CHWs’ provision of addiction-related service provision for PWUD and support of their family members; and (3) the intervention will improve family members’ support for PWUD’s engagement in the addiction continuum. The trial will be conducted in 3 regions in Vietnam. These regions represent geographical variations in the country. The trial will include 60 CHCs, randomized to either an intervention or control condition. We propose to enroll 720 PWUD and 720 family members (12 families per CHC) as well as 180 CHWs (3 per CHC). Intervention outcomes will be evaluated at baseline and every 3 months after that (Figure 2). We plan to select communes with the highest number of PWUD in each region to ensure adequate participants. The selected CHC in each region will be paired based on the number of PWUD. Within each pair, the CHC will be randomized after baseline.

#### Eligibility and Recruitment

To recruit PWUD, we will post project recruitment flyers in major community activity sites, such as local commune culture centers, hospitals or pharmacies, and other places where local PWUD usually gather. PWUD interested in the study can contact our recruitment staff using the information printed on the flyers. Recruitment staff will meet with potential participants individually to screen for eligibility. Eligibility criteria include (1) age of 18 years and older, (2) having a history of drug use, (3) having disclosed the drug-using status to at least one of the family members and being willing to invite the family member to our study, and (4) currently residing in the selected communes. Written informed consent and consent to contacting a pointed family member will be obtained.

The consented PWUD will be asked to choose a family member in the order of closeness, usually starting from spouse, mother, father, sibling, to other relatives. The inclusion criteria for a family member are (1) age of 18 years and older, (2) being an immediate or extended family of the PWUD, and (3) knowing the PWUD’s drug-using behavior. Our recruitment staff will work with the PWUD to arrange a private meeting with the nominated family member. If the nominated family member also has a history of drug use, he or she will be enrolled as a PWUD participant, and another family member will be recruited from the family. All family member participants will sign a written informed consent.

Eligibility criteria for CHWs include age of 18 years and older and being a CHW (including doctors, assistant doctors, and nurses) who regularly provides health services to community residents. Supporting staff, including cleaners, accountants, and security personnel, will be excluded. There are usually 3 to 5 eligible CHWs in each CHC, and they will be invited to participate in the study. Written informed consent will be obtained.

#### Intervention Implementation

Based on our literature review and intervention research experience, we plan to implement an intervention that combines CHWs’ in-person group training sessions, online support groups, CHW-delivered home visits, and mobile phone text messages for PWUD and their family members. The group training sessions will be conducted with participating CHWs in the intervention arm. We anticipate conducting 2 group sessions, each lasting approximately 90 minutes. The training topics will concentrate on community-based addiction service delivery. After the initial group training sessions, online support groups will be formed to facilitate continued skill building, information sharing, networking, and problem-solving. As part of the intervention requirement, trained CHWs will work as a team to deliver individualized addiction services to PWUD and family members in the community. For each participating PWUD and their family, the trained CHW team will conduct home visits to identify PWUD’s specific needs and gaps, offer referrals, and provide social and mental health support. During the home visits, activities will be conducted with family members to release the caregiver burden, enhance family support, and address other barriers. As a supplement to the home visits, mobile phone text messages will be delivered periodically from trained CHWs to PWUD participants for health promotion and service engagement. The text messages will be adaptive to individual needs and preferences.

We will collect process evaluation data to monitor and improve intervention implementation, that is, evaluator observation, intervention activity tracking, and participant self-evaluation. For the in-person group sessions, facilitators will keep records of participants’ attendance and activities conducted in each session. The investigators and field directors will observe training sessions and use a predesigned checklist to rate the adherence to session contents, time allocation, core elements, techniques, clarity, and responsiveness. For the online group discussion, the frequency and format will be tracked. Each group member’s activity, including the initiation of threads, comments, and reactions, will be captured to determine the level of engagement. In addition, all CHWs in the intervention will be given a user-friendly service log to document their experiences and feedback on the program.

#### Data Collection

The intervention assessments will consist of self-report questionnaires and medical or service records. We will collect the information at baseline and at 3-, 6-, 9-, and 12-month follow-ups. CHWs will be surveyed using the Computer-Assisted Self-Interview method, as PWUD and family members will be assessed using the Computer-Assisted Personal Interview method. We will ask PWUD participants at each assessment to donate a urine sample to test. PWUD’s service records will be chart reviewed, and their service encounters with providers, laboratory testing dates and results, and treatment status will be collected.

#### Outcome Measures

The primary outcome measure is the PWUD’s STTR fulfillment score, which is a comprehensive score that integrates medical, behavioral, and psychosocial indicators to evaluate PWUD’s health and service engagement profile. The score consists of multiple fulfillment indicators across 5 domains: Seek, Test, Treat, Retain, and Health. Most of the indicators were suggested by the STTR Data Collection and Harmonization Initiative [14,32] and adapted to the context of Vietnam. The secondary measures of interest include CHWs’ service provision, support to PWUD and their family members, and family members’ support of PWUD’s service engagement. Other relevant indicators are listed in Figure 1. All the measures will be piloted before the trial.

#### Sample Size

The study will randomize 60 CHCs (clusters) to intervention and control conditions with a 1:1 ratio. The sample size calculation was based on the primary outcome measure. We propose enrolling 720 PWUD (360 per condition) to ensure we have 80% power at 5% significance level to detect a standardized effect size of 0.41 when comparing the mean change scores of the primary outcome between intervention and control conditions. The proposed sample size accounts for the commune-level clustering, repeated observations within PWUD, and an attrition rate up to 8% for PWUD.

#### Data Analysis

All participants will be analyzed on an intent-to-treat basis. Descriptive statistics, frequencies for demographic and baseline characteristics, and all the outcome measures will be summarized at baseline by intervention condition. For each study population, generalized linear mixed-effects regression models with appropriate link functions will be used to compare the changes in the primary outcome from baseline between intervention and control groups. Each regression model will include commune- and participant-level random effects to account for dependence within communes and repeated observations within participants. The main effects included in each model are intervention condition, visit, and a condition-by-visit interaction term. Intervention effects on the primary outcomes will be estimated using model contrasts. The preselected demographic and background characteristics (covariates) will be added to evaluate whether the intervention effects on primary outcomes remain significant. The same analytical approach will be applied to each STTR domain score and the secondary outcomes of interest. We also plan to conduct several exploratory data analyses to (1) examine the intervention effect on each STTR fulfillment indicator by subgroup, (2) explore the interrelationship among PWUD’s STTR domains or indicators to identify service gap, and (3) explore the interconnectedness of intervention effects on CHW, PWUD, and family members.

#### Ethics Approval

The study protocol was reviewed and approved by the Institutional Review Board of the University of California, Los Angeles (UCLA IRB#20-001594-AM-0001) and the Institutional Review Board in Bio-Medical Research, National Institute of Hygiene & Epidemiology in Vietnam (NIHE IRB VN01057/IORG 0008555; No: NIHE IRB - 28/2022).

## Results

The intervention and assessment instruments have been developed based on the findings from formative studies (in-depth interviews and focus groups). The pilot activities are scheduled for 2023 and followed by the main trial. For the assessment of trial outcomes, we are scheduled to collect data at baseline and at 3-, 6-, 9- and 12-month follow-ups. We will distribute reports based on the baseline data in early 2024. The intervention outcome results will be available within 6 months of the final data collection date, that is, the main study findings are expected to be available by early 2025.

## Discussion

### Expected Outcomes

We anticipate the intervention to be feasible and provide community stakeholders, health professionals, and policy makers with insights for the future establishment and implementation of a community-based addiction service continuum. Community-based service delivery model has been considered a desirable strategy, and the scale-up of accessible and community-led harm reduction services is a global priority [34]. The existing primary health care system in Vietnam provided an opportunity and platform to test an intervention that features the alliance of CHWs and family members to provide a continuum of addiction services for PWUD. If tested effective, the delivery strategies in this study can potentially serve as an example to provide community-based addiction services in other countries. For example, the CHW training package can offer a transferable tool for training primary care providers in addiction disorders in a global context.

Compared to prior work on community-based service delivery models, this study focuses on community partnership rather than each target population, such as PWUD, family members, or CHWs, respectively. We anticipated that improving CHWs’ capacity through intervention training will strengthen their support for PWUD and family members, which will in turn improve addiction service engagement and retention. With the capacity building in the community, we anticipate that the trained CHWs will focus on adapting services across the care continuum to reflect different conditions of individuals and families, potentially enhancing community service delivery with sustainable outcomes.

Another strength of this study is to pay special attention to the vulnerability of family members and the role of family support. We have learned from the existing literature and previous studies that family can be mobilized to become a locally available, trustful, and sustainable support system. We anticipate that our intervention effort will form a partnership between CHWs and family members. Our intervention aims to address the impact of addiction and stigma on families and the burden and challenges faced by family members. The study findings would contribute to the growing body of literature on the role of family support in community-based addiction services and care.

This study has some limitations. First, considering that we integrate various components (eg, in-person sessions, online groups, home visits, and text messages) in the intervention, we will not have the opportunity to assess the effect of each strategy. Second, the intervention program in this study is designed to use the capacity-building approach to train CHWs. The process of training health workers to improve their knowledge and skills to enhance care and services could be too complex to be identified. Third, although the intervention aims to mobilize community health workers and PWUD’s family members to strengthen community partnerships to support addiction services, the study’s outcome assessments and measures are mainly at the individual level.

### Dissemination Plan

The dissemination of study findings will include articles in both Vietnamese and English journals, international conference presentations, and other scholarly channels. Also, we will disseminate study findings to stakeholders to support the development and implementation of health care policies in Vietnam. We will make the intervention training guides, tools, manuals, and materials available to other interested organizations and agencies.

## Appendix

Multimedia Appendix 1 Peer review report by the National Institute on Drug Abuse - Community-Level Health Promotion Study Section - Healthcare Delivery and Methodologies Integrated Review Group - Center for Scientific Review (National Institutes of Health, USA).

## Abbreviations

CHC: commune health center
CHW: community health worker
MAT: medication-assisted therapy
PWUD: people who use drugs
STTR: Seek-Test-Treat-Retain

## Data Availability

All data collected will be accessible by the study investigators, who have the right to analyze and publish data. After the publication of the primary findings, deidentified and individual-level data can be available upon reasonable requests by graduate students and research scholars. Data requests can be submitted to the investigators at both participating agencies in Vietnam and the United States.

## References

[1] World drug report 2021. United Nations.

[2] Volkow ND, Frieden TR, Hyde PS, Cha SS (2014). Medication-assisted therapies — tackling the opioid-overdose epidemic. N Engl J Med.

[3] Fairley M, Humphreys K, Joyce VR, Bounthavong M, Trafton J, Combs A, Oliva EM, Goldhaber-Fiebert JD, Asch SM, Brandeau ML, Owens DK (2021). Cost-effectiveness of treatments for opioid use disorder. JAMA Psychiatry.

[4] Oesterle TS, Kolla BP, Rummans TA, Gold MS (2020). Medication-assisted therapies for opioid use disorders in patients with chronic pain. J Neurol Sci.

[5] Bell J, Strang J (2020). Medication treatment of opioid use disorder. Biol Psychiatry.

[6] Eibl JK, Gauthier G, Pellegrini D, Daiter J, Varenbut M, Hogenbirk JC, Marsh DC (2017). The effectiveness of telemedicine-delivered opioid agonist therapy in a supervised clinical setting. Drug Alcohol Depend.

[7] Jones CM, Campopiano M, Baldwin G, McCance-Katz E (2015). National and state treatment need and capacity for opioid agonist medication-assisted treatment. Am J Public Health.

[8] Korthuis PT, McCarty D, Weimer M, Bougatsos C, Blazina I, Zakher B, Grusing S, Devine B, Chou R (2016). Primary care–based models for the treatment of opioid use disorder. Ann Intern Med.

[9] (2014). Community management of opioid overdose. World Health Organization.

[10] Amodia DS, Cano C, Eliason MJ (2005). An integral approach to substance abuse. J Psychoactive Drugs.

[11] Agonafer EP, Carson SL, Nunez V, Poole K, Hong CS, Morales M, Jara J, Hakopian S, Kenison T, Bhalla I, Cameron F, Vassar SD, Brown AF (2021). Community-based organizations' perspectives on improving health and social service integration. BMC Public Health.

[12] Riza E, Kalkman S, Coritsidis A, Koubardas S, Vassiliu S, Lazarou D, Karnaki P, Zota D, Kantzanou M, Psaltopoulou T, Linos A (2020). Community-based healthcare for migrants and refugees: a scoping literature review of best practices. Healthcare (Basel).

[13] Public policy statement: definition of addiction. ASAM (American Society of Addiction Medicine).

[14] Chandler R, Gordon MS, Kruszka B, Strand LN, Altice FL, Beckwith CG, Biggs ML, Cunningham W, Chris Delaney J, Flynn PM, Golin CE, Knight K, Kral AH, Kuo I, Lorvick J, Nance RM, Ouellet LJ, Rich JD, Sacks S, Seal D, Spaulding A, Springer SA, Taxman F, Wohl D, Young JD, Young R, Crane HM (2017). Cohort profile: seek, test, treat and retain United States criminal justice cohort. Subst Abuse Treat Prev Policy.

[15] Reichert J, Gleicher L (2019). Probation clients' barriers to access and use of opioid use disorder medications. Health Justice.

[16] Skolnick P (2018). The opioid epidemic: crisis and solutions. Annu Rev Pharmacol Toxicol.

[17] Dowell D, Haegerich TM, Chou R (2016). CDC guideline for prescribing opioids for chronic pain--United States, 2016. JAMA.

[18] Lagisetty P, Klasa K, Bush C, Heisler M, Chopra V, Bohnert A (2017). Primary care models for treating opioid use disorders: What actually works? A systematic review. PLoS One.

[19] (2008). Strengthening commune health centers in Vietnam: assessing the impact of the Atlantic Philanthropies 2008-16. SSCRC.

[20] Tran B, Mai H, Fleming M, Do H, Nguyen T, Vuong Q, Ho M, Van Dam N, Vuong T, Ha G, Truong N, Latkin C, Ho C, Ho R (2018). Factors associated with substance use and sexual behavior among drug users in three mountainous provinces of Vietnam. Int J Environ Res Public Health.

[21] (2019). UNAIDS Data.

[22] (2015). Community-based drug treatment models for people who use drugs. Harm Reduction International.

[23] (2016). Decentralization of HIV testing services to increase access for people who inject drugs in Viet Nam. UNAIDS.

[24] Lin C, Tuan NA, Li L (2018). Commune health workers' methadone maintenance treatment (MMT) knowledge and perceived difficulties providing decentralized MMT services in Vietnam. Subst Use Misuse.

[25] Khue PM, Tham NT, Thanh Mai DT, Thuc PV, Thuc VM, Han PV, Lindan C (2017). A longitudinal and case-control study of dropout among drug users in methadone maintenance treatment in Haiphong, Vietnam. Harm Reduct J.

[26] Nguyen LH, Nguyen HTT, Nguyen HLT, Tran BX, Latkin CA (2017). Adherence to methadone maintenance treatment and associated factors among patients in Vietnamese mountainside areas. Subst Abuse Treat Prev Policy.

[27] Pham LTT, Kitamura A, Do HM, Lai KA, Le NT, Nguyen VTT, Kato M (2017). Retrospective analysis of antiretroviral therapy uptake and retention of male clients receiving methadone maintenance therapy in two provinces in Vietnam: potential synergy of the two therapies. Harm Reduct J.

[28] Bagley SM, Peterson J, Cheng DM, Jose C, Quinn E, O'Connor PG, Walley AY (2015). Overdose education and naloxone rescue kits for family members of individuals who use opioids: characteristics, motivations, and naloxone use. Subst Abus.

[29] Ventura A, Bagley S (2017). To improve substance use disorder prevention, treatment and recovery: engage the family. J Addict Med.

[30] Li L, Tuan NA, Liang L, Lin C, Farmer SC, Flore M (2013). Mental health and family relations among people who inject drugs and their family members in Vietnam. Int J Drug Policy.

[31] Salter ML, Go VF, Minh NL, Gregowski A, Ha TV, Rudolph A, Latkin C, Celentano DD, Quan VM (2010). Influence of perceived secondary stigma and family on the response to HIV infection among injection drug users in Vietnam. AIDS Educ Prev.

[32] Seek, Test, Treat and Retain. National Institute on Drug Abuse.

[33] Chandler RK, Kahana SY, Fletcher B, Jones D, Finger MS, Aklin WM, Hamill K, Webb C (2015). Data collection and harmonization in HIV research: the Seek, test, treat, and retain initiative at the national institute on drug abuse. Am J Public Health.

[34] The global state of harm reduction 2022 8th edition. Harm Reduction International.

