# Supplementary material for: Strengthening Addiction Care Continuum Through Community Consortium in Vietnam: Protocol for a Cluster-Randomized Controlled Trial
Source: JMIR Res Protoc. 2023 Mar 22;12:e44219. doi: 10.2196/44219 (PMC10131887; doi:10.2196/44219)
Supplement: Multimedia Appendix 1 [file resprot_v12i1e44219_app1.pdf]

1R01DA050678-01A1 Li, Li

PROTECTION OF HUMAN SUBJECTS UNACCEPTABLE

RESUME AND SUMMARY OF DISCUSSION: This study aims to develop and test the feasibility of an intervention to enhance the addiction service continuum with the joint effort of commune health workers (CHW) and family members of people who use opioids (PWUO) in Vietnam. The impact of the intervention will also be evaluated through a randomized controlled trial. The resubmission retains some strong features of the previous submission, including the significant focus on targeting opioid users, the experienced investigative team with a collaboration history, the outstanding research environment, the intervention being guided by the Anderson's behavioral model, the inclusion of diverse focus groups, and the solid study design. The revised application is responsive to most critiques from prior review, including more discussion about the unique contribution of the proposed study and better explained study measures. During discussion, some weaknesses were identified. The study hypotheses can be more clearly presented. More details about the text messaging are needed. The study attrition rate used in the power analysis seems low. Overall, however, the Committee agreed that the identified weaknesses are minor. This is a significant application focusing on opioid addiction, an international issue, and the enthusiasm was high.

DESCRIPTION (provided by applicant): The current opioid epidemic calls for global attention. Opioid use disorder is a chronical condition that requires long-term comprehensive health services, from assessment, treatment, continuous monitoring, to extended care. Medication-assisted therapy, although available as an effective strategy to treat opioid addiction, has been significantly underutilized due to the hard-to-reach nature of people who use opioids (PWUO) and the shortage of addiction specialist. An impetus for expanding and enhancing addiction treatment is to mobilize community-based healthcare agencies and family members of PWUO. The study will take advantage of the existing community health care infrastructure and family support systems in Vietnam to develop and test an intervention to strengthen a continuum of addiction services. The intervention, entitled "Community Care Consortium (CCC)," features community health workers' joint effort with family members to provide patient-centered, individualized addiction care and support. The intervention will be developed and tested through three phases in three regions of Vietnam (Ninh Binh, Da Nang, and Can Tho). In Phase 1, we will conduct formative studies with community health workers, community representatives, PWUO, and their family members to identify barriers to addiction service utilization and discuss potential strategies to establish a continuum of addiction services. Based on the formative study findings, the CCC intervention and its implementation plans will be developed through workgroup meetings with researchers, community members, and target users. In Phase 2, the CCC Intervention will be piloted in three communes and revised based on acceptability/feasibility data, process evaluation, and feedback from field staff and participants. In Phase 3, a randomized controlled trial of the CCC Intervention will be conducted in 60 communes, which will be randomized to either an intervention condition or a control condition (30 in each condition). A total of 720 PWUO, 720 of their family members, and 180 commune health workers (CHW) will participate in the study. The intervention outcomes on PWUO, CHW, and family members will be assessed with the data collected at baseline, 3-, 6-, 9- and 12-month follow-ups.

PUBLIC HEALTH RELEVANCE: The significant gaps in people who use opioids (PWUO)'s care seeking, testing, treatment, and retention are impeding efforts to address the current opioid epidemic. This study aims to mobilize community health workers and PWUO's family members to support a continuum of addiction services in Vietnam. Study findings are expected to demonstrate a model of community-based addiction service continuum that can be transferable to other countries, including the U.S.

CRITIQUE 1

Significance: 2
Investigator(s): 1
Innovation: 2
Approach: 2
Environment: 1

Overall Impact: Medication-assisted therapy has been significantly underutilized due to the hard-to-reach nature of people who use opioids (PWUO) and the shortage of addiction specialist. This resubmission application will take advantage of the existing community health care infrastructure and family support systems in Vietnam to develop and test an intervention to strengthen a continuum of addiction services. The intervention, entitled "Community Care Consortium (CCC)," features community health workers' joint effort with family members to provide patient-centered, individualized addiction care and support. The intervention will be developed and tested through three phases in three regions of Vietnam (Ninh Binh, Da Nang, and Can Tho). Phase 1 involves formative studies with community health workers, community representatives, PWUO, and their family members to identify barriers to addiction service utilization and discuss potential strategies to establish a continuum of addiction services. Based on the formative study findings, the CCC intervention and its implementation plans will be developed through workgroup meetings with researchers, community members, and target users. In Phase 2, the CCC Intervention will be piloted in three communes and revised based on acceptability/feasibility data, process evaluation, and feedback from field staff and participants. In Phase 3, an RCT of the CCC Intervention will be conducted in 60 communes (randomized to either an intervention condition or a control condition; 30 in each condition). A total of 720 PWUO, 720 of their family members, and 180 commune health workers (CHW) will participate in the study. The intervention outcomes on PWUO, CHW, and family members will be assessed with the data collected at baseline, 3-, 6-, 9- and 12-month follow-ups. This proposal is strong with regard to investigators, environment, and innovation. The research team was highly responsive to the prior set of reviews, particularly with regard to clarifying how this work is distinct from other work conducted by the team, clarifying ambiguities related to recruitment and its feasibility, further specifying and justifying some measures used, and articulating what the formative research entails and how it is used.

1. Significance

Strengths

- The proposed study has the potential of having high impact at the population level and is very timely and important.
- The research team provides a compelling scientific premise and rationale for the goals of the study in general and for the methods employed.

Weaknesses

- No particularly notable weaknesses.

2. Investigator(s)

Strengths

- As stated previously, the study team is comprised of PI Dr. Li who has an impressive track record and well-suited expertise; Dr. Li is supported by an exemplary group of co-investigators, including the in-country PI Dr. Nguyen, Head of the HIV/AIDS Division at National Institute of Hygiene and Epidemiology (NIHE) in Vietnam. Other team members bring expertise in implementation science (Dr. Li), biostatistics (Dr. Liang), addiction (Dr. Shoptaw), and other

areas. The team has a track record of work together. The specific roles and areas of contribution among team members are clear.

Weaknesses

- None noted.

3. Innovation

Strengths

- The study is novel in that it aims to 1) establish a locally available and sustainable support system to provide a continuum of addiction services; 2) construct a single composite score to reflect individual opioid users' stance in a continuum of care; and 3) provide patient-centered differentiated care for opioid users and family members with various special needs.

Weaknesses

- No particularly notable weaknesses.

4. Approach

Strengths

- The research team was highly responsive to the prior set of reviews, particularly with regard to clarifying how this work is distinct from other work conducted by the team, clarifying ambiguities related to recruitment and its feasibility, further specifying and justifying some measures used, and articulating what the formative research entails and how it is used.
- Sex as a biological variable is addressed.
- Rigor and reproducibility addressed.

Weaknesses

- No particularly notable weaknesses.

5. Environment

Strengths

- The research environment is well-suited to conduct this research. In particular, the longstanding research team and institutional history of collaboration provide a solid foundation.

Weaknesses

- None noted.

Study Timeline

Strengths

- Feasible timeline.

Weaknesses

- None noted.

Protections for Human Subjects

Acceptable Risks and/or Adequate Protections

- Appropriate considerations.

Data and Safety Monitoring Plan (Applicable for Clinical Trials Only):

Acceptable

- Appropriate considerations.

Inclusion Plans

- Sex/Gender: Distribution justified scientifically
- Race/Ethnicity: Distribution justified scientifically
- For NIH-Defined Phase III trials, Plans for valid design and analysis: Not applicable
- Inclusion/Exclusion Based on Age: Distribution justified scientifically
- Appropriate.

Vertebrate Animals

Not Applicable (No Vertebrate Animals)

Biohazards

Not Applicable (No Biohazards)

Resubmission

- The research team was highly responsive to the prior set of reviews, particularly with regard to clarifying how this work is distinct from other work conducted by the team, clarifying ambiguities related to recruitment and its feasibility, further specifying and justifying some measures used, and articulating what the formative research entails and how it is used.

Applications from Foreign Organizations

Justified

- Well considered/justified.

Resource Sharing Plans

Acceptable

Authentication of Key Biological and/or Chemical Resources

Not Applicable (No Relevant Resources)

Budget and Period of Support

Recommend as Requested

CRITIQUE 2

Significance: 1
Investigator(s): 1
Innovation: 3
Approach: 2
Environment: 1

Overall Impact: This resubmission aims to use the grassroots system of healthcare in Vietnam of Commune Health Centers and Community Health Workers (CHW) to develop and conduct a community-based service delivery intervention to enhance the addiction service continuum for people who use opioids (PWUO). The well thought-out plan starts with formative interviews with key stakeholders to inform potential strategies, workgroup meetings to develop the intervention, a pilot test of the intervention, and an RCT to test the intervention on a larger scale. They wisely will measure intervention effectiveness for both PWUO (measured using their novel composite score) as well as families and CHW. The team has a long track record of collaboration between UCLA and Vietnam, which will increase the chance of success for this ambitious proposal. Other strengths include harnessing multiple resources along the continuum of care, particularly the families of users and community health workers, and adapting/using a number of evidence-based interventions. Overall, this is a very significant project that has been well designed to impact PWUO along the entire continuum of care by leveraging community resources, with potential to have an impact both in Vietnam and the United States.

1. Significance

Strengths

- Community approach to moving PWUO along the continuum of care, which helps with the management of integrated care along the entire continuum as well as improving clinical outcomes. This approach has been underutilized and has the potential to greatly improve outcomes.
- Using both CHWs and family members as intervention partners can help build a less stigmatized, more respectful way to provide addiction care support. In Vietnam, there are currently challenges (particularly stigmatizing attitudes of many CHWs) to providing addiction care, and this proposed intervention will directly impact these attitudes.
- A number of existing evidence-based interventions along the entire continuum of care already exist; by adapting/using these, the feasibility of a successful intervention plan is increased.

Weaknesses

- None noted.

2. Investigator(s)

Strengths

- The research partnership between the Key Personnel is very strong, and includes partners both in Vietnam and in the United States. They have been very productive and effective in their prior collaborative projects.
- PI Li is well positioned to serve as PI. She has 20+ years of experience studying substance use in the U.S., China, and Vietnam and has served as PI on prior NIH-funded projects to support people living with HIV in Vietnam and to enhance the role of CHW in HIV and drug abuse

prevention in Vietnam. She has widely published on intervention studies, in Vietnam and elsewhere, to reduce drug abuse.

- The lead in Vietnam, Co-I Tuan, has implemented many national interventions in Vietnam, some in partnership with researchers in the United States.

Weaknesses

- None noted.

3. Innovation

Strengths

- Measure of each PWUO on the continuum of care allows for an overall assessment of each individual; combined with domain-specific scores, this will allow CHW to provide targeted services during the intervention. This is a novel and potentially very powerful approach to providing targeted addiction services.
- Including both CHWs and family members is novel and has the potential to increase PWUO's quality of addiction services.

Weaknesses

- The innovation is driven by the delivery of services; the interventions themselves are not novel.

4. Approach

Strengths

- The timeline and ordering of activities are appropriate: Develop, then pilot test, then conduct an RCT of the intervention.
- Focus groups and in-depth interviews (Aim 1) will include a diverse group of 4 types of key stakeholders, including CHW, community representatives (NGO leaders/healthcare administrators, etc.), PWUOs, and family members.
- The development process itself will use a community-based participatory approach using intervention mapping, with a multidisciplinary workgroup to identify intervention opportunities, tailor the intervention to the local conditions, and develop an implementation and evaluation plan. Team has successfully developed other interventions using this approach. If the focus group/in depth interviews are not sufficient, the team is also conducting a literature review of other intervention strategies.
- Pilot includes communes/CHW/PWUO from each of the three study sites. Process data will be collected and they will conduct a feasibility/acceptability evaluation to prepare for the larger RCT. These plans are appropriate for scaling up from a pilot to larger RCT.
- Participant recruitment through site-based local advertisements and referrals from other PWUOs. They will increase participant retention through gathering information on tracking forms. This is a good plan which they have previously successfully implemented in other studies on PWUO in the same area.
- The potential interventions focus on a wide range of services along the continuum of care.

Weaknesses

- Unclear how any discrepancies/different perspectives uncovered between groups in the focus groups will be resolved.

- They state that they will consider distances between communes to avoid contamination, but there are no details about how this will work. How far apart will they need to be to avoid contamination?
- Power analysis depends on low study attrition (less than 10%).

5. Environment

Strengths

- UCLA provides a strong environment for study team in the US, where there is ongoing NIH-funded research being conducted internationally on HIV and substance use amongst PWUO.
- NIHE Vietnam provides a complimentary and essential environment with appropriate government supports to successfully implement the intervention in Vietnam.

Weaknesses

- None noted.

Study Timeline

Strengths

- Study activities even spread out and feasible in a 5 year time span.

Weaknesses

- None noted.

Protections for Human Subjects

Acceptable Risks and/or Adequate Protections

- Adequate protections to keep confidentiality
- Steps taken to avoid coercion of participation by PWUO

Data and Safety Monitoring Plan (Applicable for Clinical Trials Only):

Acceptable

- o DSMB with investigators from UCLA and Vietnam will oversee protocol and intervention.
- o PI responsible for integrity of research procedures.

Inclusion Plans

- Sex/Gender: Distribution justified scientifically
- Race/Ethnicity: Distribution justified scientifically
- For NIH-Defined Phase III trials, Plans for valid design and analysis: Not applicable
- Inclusion/Exclusion Based on Age: Distribution justified scientifically
- All participants must be 18+; gender distribution will represent %s in Vietnam of PWUO and CHW; all participants are Asian (Vietnamese).

Vertebrate Animals

Not Applicable (No Vertebrate Animals)

Biohazards

Not Applicable (No Biohazards)

Resubmission

- Very responsive resubmission: Key changes include shifting Aim 2 to not be dependent on the success of Aim 1; refining the recruitment procedures; emphasizing what is new/unique about this project vs. prior projects (this one specifically targets PWUO and focuses on training CHW); and demonstrating the external validity of the project to locations in the United States and elsewhere.

Applications from Foreign Organizations

Justified

- Vietnam provides a unique opportunity to take advantage of the existing grassroots health system in a setting with a high burden of opioid addiction.
- The study team has extensive experience collaborating between the U.S. and Vietnam.

Resource Sharing Plans

Acceptable

- After data are de-identified, cleaned, and validated, and main findings are published, study team will make study data available upon request with the scientific community to those who make a direct request to the PI and indicate the data will be used for research.

Authentication of Key Biological and/or Chemical Resources

Not Applicable (No Relevant Resources)

Budget and Period of Support

Recommend as Requested

CRITIQUE 3

Significance: 3
Investigator(s): 2
Innovation: 3
Approach: 4
Environment: 2

Overall Impact: This resubmitted R01 application seeks to develop an intervention to enhance the addiction service continuum for opioid users in Vietnam and evaluate it in a three-region randomized-controlled trial (RCT). The intervention, Community Care Consortium (CCC), involves commune health workers (CHW) in commune health centers (CHC) and family members in medication-assisted treatment (MAT) for people who use opioids (PUOW). The application has been responsive to the prior review though some previously identified weaknesses. The investigative team and environments are

very strong. Some aspects of the approach are confusing. Significance, innovation, and approach are hampered by a general lack of emphasis on the science of the proposal. Hypotheses are not explicitly stated, nor does the application inform how it will address weaknesses in prior research or contribute to the literature.

1. Significance

Strengths

- This project seeks to address gaps in the care continuum for PWUO, which impede efforts to stem the opioid epidemic.
- It is anticipated that results will be generalizable to other countries with rural and remote populations, including the US.
- The prior research that serves as the key support for the proposed project is rigorous.

Weaknesses

- It remains somewhat unclear how the proposed project will contribute to the literature. The application does not explicitly identify weaknesses in the rigor of prior research that the proposed project seeks to address.

2. Investigator(s)

Strengths

- PI Li has assembled a strong team with complementary expertise and valuable prior experience in Vietnam and with RCTs related to injection drug use and HIV. She has a strong record of research productivity and a history of collaboration with the other members of the investigative team.
- Co-I Lin brings expertise in epidemiology and implementation science.
- Co-I Liang brings expertise in biostatistics.
- Co-I Shoptaw brings expertise in behavioral and biomedical treatment of HIV and substance use disorders.
- In-country PI Nguyen is former head of the HIV/AIDS Division at National Institute of Hygiene and Epidemiology (NIHE) in Vietnam. He has experience implementing national programs, including the President's Emergency Plan for AIDS Relief.

Weaknesses

- According to her personal statement, co-I Lin expects to be co-PI on the proposed study but is not designated as such.

3. Innovation

Strengths

- The proposed project is innovative in using an individual-level composite STTR score to evaluate intervention outcomes on individual PWUO.
- It is also innovative to use individual-level scores on STTR indices to guide care.
- Engaging families is somewhat innovative.

Weaknesses

- As the application builds its case for innovation around the STTR score, it does not explain how the proposed project innovates in other ways.

4. Approach

Strengths

- The UCLA Semel Institute, Center for Community Health and the Vietnam National Institute of Hygiene and Epidemiology have collaborated since 2010 on community-based projects targeting PWUO in Vietnam.
- The approach is supported by several previous studies conducted by the investigative team.
- The approach uses NIDA's "seek, test, treat, and retain" (STTR) model of the addiction services continuum.
- The research methodology promotes a robust and unbiased approach. The application lays out a three-phase plan with formative research, pilot-testing, and a multi-site RCT in three regions of Vietnam.
- CCC will use existing evidence-based methods to train CHW to reduce misconceptions about MAT and stigmatizing attitudes towards PWUO and foster family support for treatment-seeking and adherence.
- Process monitoring and evaluation is described.
- Data collection includes a 12-month follow-up to gauge durability of effects.
- STTR score construction is described.
- The power analysis is appropriate.
- Letters of support are provided from each region and the National Institute of Hygiene and Epidemiology.

Weaknesses

- Hypotheses are not explicitly articulated. While the data analysis plan includes testing for intervention effects on primary and secondary outcomes and subgroup analysis, and while individual characteristics will be included in the analyses, there are no hypothesized moderators or mediators of intervention effectiveness.
- The plan for the intervention is confusing. Components for CHW and components for PWUOs are described in a conflated manner. Online support groups appear targeted to the CHW while text messages appear targeted to the PWUOs by the CHWs, but this is difficult to parse.
- Details regarding the text messaging are lacking. It is unclear whether CHW will have to send SMS messages manually or whether the process will be automated. If the former, it would seem burdensome to the CHWs. If the latter, the platform for SMS message distribution is not described.
- The approach does not address potential problems/pitfalls and alternative strategies.

5. Environment

Strengths

- The facilities and other resources for UCLA Semel Institute, Center for Community Health and the Vietnam National Institute of Hygiene and Epidemiology are more than sufficient to conduct the proposed work.

- The foreign justification is sound.
- The choice of three different regions in Vietnam for the cluster randomized trial enhances external validity and generalizability.

Weaknesses

- No major weaknesses noted.

Study Timeline

Strengths

- The study timeline includes important milestones.
- The timeframe for achieving study milestones seems reasonable.

Weaknesses

- No major weaknesses noted.

Protections for Human Subjects

Unacceptable Risks and/or Inadequate Protections

- The human subjects section does not define adverse events or specify how serious adverse events such as overdose will be addressed.

Data and Safety Monitoring Plan (Applicable for Clinical Trials Only):

Unacceptable

- o The DSMP does not adequately address trial safety and reportable events.

Inclusion Plans

- Sex/Gender: Distribution justified scientifically
- Race/Ethnicity: Distribution justified scientifically
- For NIH-Defined Phase III trials, Plans for valid design and analysis: Not applicable
- Inclusion/Exclusion Based on Age: Distribution justified scientifically
- Inclusions are based on sex/gender and race/ethnicity profiles of study populations. Children are appropriately excluded.

Vertebrate Animals

Not Applicable (No Vertebrate Animals)

Biohazards

Not Applicable (No Biohazards)

Resubmission

- The application was largely responsive to the prior review, as described in the introduction. However, some previously identified weaknesses remain.

Resource Sharing Plans

Acceptable

Authentication of Key Biological and/or Chemical Resources

Not Applicable (No Relevant Resources)

Budget and Period of Support

Recommend as Requested

THE FOLLOWING SECTIONS WERE PREPARED BY THE SCIENTIFIC REVIEW OFFICER TO SUMMARIZE THE OUTCOME OF DISCUSSIONS OF THE REVIEW COMMITTEE, OR REVIEWERS' WRITTEN CRITIQUES, ON THE FOLLOWING ISSUES:

PROTECTION OF HUMAN SUBJECTS: UNACCEPTABLE

- Adverse events need to be better addressed in the protection of human subjects section and in the Data and Safety Monitoring Plan (see Critique 3).

INCLUSION OF WOMEN PLAN: ACCEPTABLE

INCLUSION OF MINORITIES PLAN: ACCEPTABLE

INCLUSION ACROSS THE LIFESPAN: ACCEPTABLE

COMMITTEE BUDGET RECOMMENDATIONS: The budget was recommended as requested.

Footnotes for 1 R01 DA050678-01A1; PI Name: Li, Li

NIH has modified its policy regarding the receipt of resubmissions (amended applications).See Guide Notice NOT-OD-18-197 at https://grants.nih.gov/grants/guide/notice-files/NOT-OD-18-197.html. The impact/priority score is calculated after discussion of an application by averaging the overall scores (1-9) given by all voting reviewers on the committee and multiplying by 10. The criterion scores are submitted prior to the meeting by the individual reviewers assigned to an application, and are not discussed specifically at the review meeting or calculated into the overall impact score. Some applications also receive a percentile ranking. For details on the review process, see http://grants.nih.gov/grants/peer_review_process.htm#scoring.
